# The Roosevelt Hospital

**Published:** 1900-04-28

**Authors:** 


### THE ROOSEVELT HOSPITAL.

In consequence of the resignation of Dr. McBurney from the staff of the Roosevelt Hospital in New York, Dr. R. F. Weir and Dr. W. T. Bull have been appointed visiting surgeons. Thus came to an end a very interesting experiment in hospital management. According to the New York *Medical Record*, the managers of the Roosevelt Hospital some 18 years ago established a continuous surgical service under a single head, and this post Dr. McBurney has held for the last dozen years, making it the most notable surgical service in the country. The condition of affairs at this hospital seems to have been peculiar, not merely in regard to the size of the surgical clinic, but in the fact of the control and influence of a single chief extending down through all the departments, and of the consequent intimate relation between the in-patient and the out-patient services. No one can doubt the importance of the one-man control system, both in regard to the working of a hospital, the making of a school, and the training of assistants. The difficulty is to find a succession of men who can properly fill such a post.

